# Suicide among post-Arabellion refugees in Germany

**DOI:** 10.1192/bjo.2024.755

**Published:** 2024-10-25

**Authors:** Nensy Thu Ha Le, Jon Genuneit, Gerald Brennecke, Georg von Polier, Lars White, Daniel Radeloff

**Affiliations:** Department of Child and Adolescent Psychiatry, Psychotherapy and Psychosomatics, Medical Faculty, Leipzig University, Leipzig, Germany; Paediatric Epidemiology, Department of Paediatrics, Medical Faculty, Leipzig University, Leipzig, Germany; Department of Child and Adolescent Psychiatry, Psychotherapy and Psychosomatics, Medical Faculty, Leipzig University, Leipzig, Germany; and Institute of Legal Medicine, University Medicine Halle (Saale), Halle (Saale), Germany

**Keywords:** Suicide, immigration, flight, Arabellion and Arab Spring, Germany

## Abstract

**Background:**

Although immigrants are considered to be vulnerable to mental illness, there is limited knowledge regarding their suicide mortality.

**Aims:**

To investigate standardised mortality ratios (SMR) for suicide among the largest immigrant populations in Germany before and after the refugee movement of 2015.

**Method:**

Data on immigrants and the general population in Germany between 2000 and 2020 were provided by the scientific section of the Federal Statistical Office. SMR with 95% confidence intervals were calculated by indirect standardisation for gender, age and calendar year for the pre-2015 and post-2015 time interval, first for all the immigrant populations studied and second for the Syrian, Afghan and Iraqi populations separately.

**Results:**

Immigrants from the countries studied showed a lower suicide risk compared with the German reference population (SMR = 0.38, 95% CI = 0.35–0.41). No differences in SMR were found between pre- and post-2015 time intervals, in either the aggregate data for all populations or the data for Syrian, Afghan and Iraqi populations. Post-2015, Afghan immigrants (SMR = 0.68, 95% CI = 0.54–0.83) showed a higher SMR than Syrians (SMR = 0.30, 95% CI = 0.25–0.36) or Iraqis (SMR = 0.37, 95% CI = 0.26–0.48).

**Conclusions:**

Despite the many and varied stresses associated with flight, comparison of the pre- and post-2015 time intervals showed that the suicide risk of the populations studied did not change and was considerably lower than that of the German reference population. We attribute this to lower suicide rates in the countries of origin but also to flight-related selection processes that favour more resilient individuals.

‘We are witnessing a changed reality in that forced displacement nowadays is not only vastly more widespread but is simply no longer a short-term and temporary phenomenon’.^1^ Conflicts, violence, fear of persecution and human rights violations are forcing more and more people around the world to flee their homes. The number of refugees has doubled over the past decade, reaching the milestone of more than 108 million refugees by the end of 2022.^2^ The vast movement of displaced people in 2015 – most of them fleeing the military conflict in Syria – was the largest migration movement in Europe since the Second World War.^3^ Germany has been the largest European host country since 2015, and as of 2022 it is the fourth-largest in the world, having admitted 2.1 million asylum seekers and refugees.^2^ Of those who have fled to Germany, the majority of people are from Syria (approx. 674 000), followed by Afghanistan (approx. 286 000) and Iraq (approx. 211 000).^4^

Trauma exposures before and during flight are common among refugees. About 85% of Syrian refugees arriving in Germany have experienced at least one traumatic event^5^ related to war and grief resulting from multiple losses, including loss of their loved ones and homeland. In addition, they face post-migration stressors,^6^ perceived discrimination,^7^ economic hardships and prolonged uncertainty regarding their asylum status.^8^ These conflict-related and post-migration stressors are correlated with heightened risks of anxiety, depression and post-traumatic stress disorder (PTSD).^9^ German studies have begun to investigate the mental health of refugees from Syria.^10^ In line with international studies, prevalence rates are heterogeneous and range from 14% to 61% for depressive symptoms, 13% to 52% for generalised anxiety disorder and 14% to 42% for PTSD.^6^ Between 31% and 75% of the asylum seekers and refugees exceeded cut-offs for a mental disorder.^5,6^ Mental distress and psychiatric disorders seem stable after arrival. In a 1.5-year follow up study, 37% of the participants still screened positive for at least one psychiatric diagnosis.^11^ Collectively, the data suggest that asylum seekers and refugees have a substantially higher prevalence of mental disorders and are exposed to a greater number of risk factors compared with the general German population and non-refugee populations.^12^ These findings indicate that asylum seekers and refugees are high-risk groups, highlighting the relevance of addressing their mental health needs within the healthcare sector.

Mental disorders are among the strongest predictors of suicides and suicide attempts. Nevertheless, suicide rates among asylum seekers and refugees have been poorly studied, although 30% of newly arrived refugees report suicidal ideation upon arrival in Germany.^13^ Suicidal ideation in newly arrived refugees is associated with symptoms of PTSD, anxiety and depression, as well as somatic symptoms and experiences of sexual violence. Other characteristics related to sociodemographics and the refugee experience, such as longer duration of flight and being of younger age, also appear to play a significant part.^13,14^ According to a recently published meta-analysis,^15^ the overall prevalence of suicide attempts in refugees was 0.57%.

Evidence from Scandinavia reveals lower or comparable risk of suicides and suicide attempts among refugees compared with the native-born population of their country of arrival.^16–18^ In an analysis of the high-risk group of people with mental disorders, refugees mostly showed lower risk of suicide and suicide attempts across different mental disorders in comparison with the Swedish-born group.^19^ After decades of resettlement, the suicide risk for non-refugee migrants and refugees converged with the risk of the native (Swedish) population, in line with acculturation.^20^

To the best of our knowledge, this is the first study to examine suicide rates among post-Arabellion refugees living in Germany. Accordingly, we focused on refugees whose countries of origin were/are affected by the Arab Spring and made up the majority of the refugee wave in 2015 and the following years, especially Syria, Afghanistan and Iraq. We aim to compare their suicide rates with those of (a) the German population and (b) migrants from the same country of origin who immigrated before the refugee wave in 2015.

## Method

### Data acquisition

#### Number of suicide deaths and number of persons in the general population

This historical cohort study analyses all suicide deaths in Germany during the study period 2000 to 2020. Suicides were distinguished by citizenship of the deceased and related to the size of each population. We focused on populations whose countries of origin contributed significantly to the refugee movement to Germany in 2015. Data from Afghan, Syrian, Iraqi, Algerian, Moroccan, Egyptian, Tunisian, Yemeni and Jordanian populations were aggregated (populations of interest; POI) and compared with the German reference population. Syrian, Afghan and Iraqi populations represented by far the largest refugee groups and were analysed separately as well.

General population annual suicides were obtained from the Research Data Centre of the Statistical Offices of the Federal States (*Forschungsdatenzentrum der Statistischen Landesämter*, Duesseldorf, Germany). Suicides of Germans were stratified for year, gender and age group (0–14, 15–19, 20–24, …, 80–84, 85+ years).

The level of detail for the non-German populations was lower than for Germans. This was owing to the requirement of the research data centres to exclude the possibility that data could be traced; accordingly, data entries <4 are censored. Suicide counts for the POI were stratified for years only. Suicide counts for the three largest refugee populations were stratified for the years 2015 to 2020 and aggregated for the years 2000 to 2014 (owing to small population sizes and low suicide counts in the latter time period). There was no differentiation by gender or age owing to data protection requirements.

Annual census data were provided by the Federal Statistical Office Germany,^21^ stratified for year, gender, age group (0–14, 15–19, 20–24, …, 80–84, 85+ years) and nationality. The study used annual population data reported on 31 December of each year. From 2015 onwards, there was a sharp increase in the POI during each year, so the number of person-years for each year could only be approximated using the annual population data. In the absence of census data during the year, we assumed a linear increase in the POI between census dates for simplicity. Accordingly, we calculated the population for each index year as the average of the previous year's population and the index year. For details, see Supplementary material 1 available at https://doi.org/10.1192/bjo.2024.755.

#### Citizenship

After 8 years of legal residence in Germany, non-Germans have the right to become naturalised, with a shorter minimum period of residence for spouses. Children born in Germany to non-German parents automatically acquire German citizenship if one parent has been legally resident in Germany for at least 8 years. Since 2014, dual/multiple citizenship has also been possible in Germany for non-European Union citizens. According to definitions used by the statistical offices, individuals with German citizenship were considered to be German, regardless of the type of citizenship (single or dual/multiple citizenship).

The group assignments were based on citizenship because information on migration or refugee experience was not available. This is a simplification assuming that the majority of individuals from Syria, Iraq and Afghanistan in the time period 2015+ had experience of flight.

### Statistical methods

**Fig. 1:**
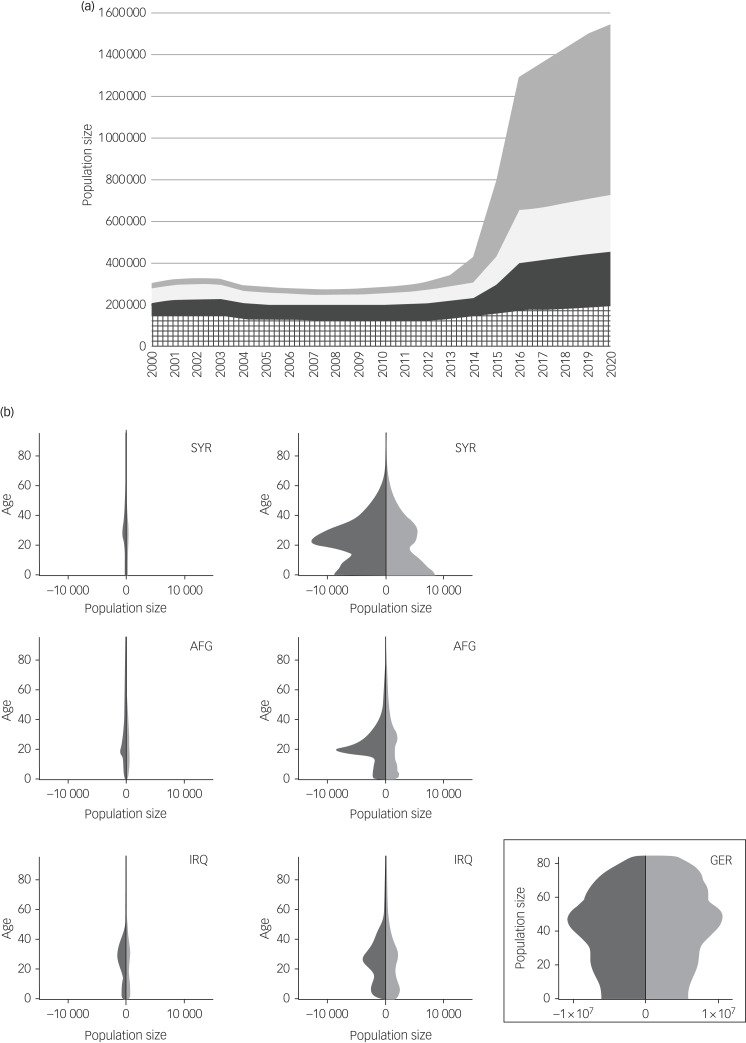
(a) Time course of the population size of Syrians (grey), Afghans (white), Iraqis (dark grey) and other populations of interest (patterned). (b) Demographics of Syrian (SYR), Afghan (AFG) and Iraqi (IRQ) populations in Germany before (left) and after (middle) the refugee movement in 2015. Dark grey indicates males; light grey indicates females. GER, German reference population (2000–2020). In both parts, the figures are based on census data published on 31 December of the respective index year.

Data were analysed using SPSS (version 29.0.0.0). Our aim was to compare the risk of suicide among the POI in Germany before and after 2015. As the demography of these populations differed extensively from the German general population (Fig. 1(a)) and changed during the study period (Fig. 1(b)), standardised mortality ratios (SMR) for suicide were calculated. An SMR quantifies the increase (or decrease) in mortality of a study population compared with a reference population, taking different age and gender distributions in these populations into account.

We opted for indirect standardisation of the data to obtain the greatest possible level of detail within the framework of data protection regulations. The total German population (2000–2020) was defined as the reference population. Expected gender-specific suicide deaths of each age group were calculated stratified for gender by multiplying age-specific suicide rates of the reference population by the number of person years in non-German populations for the specific age group and time period (2000–2014, 2015–2020). The total sum of expected deaths over genders and all age groups was calculated and compared with the total sum of observed suicide deaths in non-German populations with SMR = (observed deaths)/(expected deaths). Confidence intervals were calculated using an approximation of 95% CI = SMR ± (1.96 × s.e.), where s.e. = SMR/√(observed deaths).^21^ The true value of a normally distributed variable lies within the associated 95% confidence interval with a likelihood of error of ≤5%.

As the SMR of a reference population corresponds to 1, the SMR of the index population differs from the reference population if its 95% confidence interval does not include the value 1. When comparing two index populations (e.g. POI pre-2015 versus POI post-2015, or Afghan population post-2015 versus Iraqi population post-2015), the corresponding SMR differ if the associated 95% confidence intervals do not overlap.

During indirect standardisation, age-specific suicide rates were calculated for the reference population but not for the POI. To obtain age-specific suicide risks, we calculated suicide rates of the POI for age groups 0–29, 30–39, 40–49 and 50+ years.

#### Ethics

The study was approved by the ethics committee of the medical faculty (ID 141/18-ek) of the University of Leipzig, Germany, and conducted in accordance with the Declaration of Helsinki.

## Results

### Aggregated POI

During the survey period, 462 suicides were recorded among the POI (pre-2015: 180; post-2015: 282) and 210 895 among Germans (pre-2015: 154 101; post-2015: 56 794). In the study period, 12 575 162 life years of the POI (pre-2015: 4 649 093; post-2015: 7 926 069) and 1 558 399 145 life years of Germans (pre-2015: 1 120 191 684; post-2015: 438 207 461) were recorded. This corresponds to an unstandardised suicide rate (suicides per 100 000 life years) of 3.7 among the POI (pre-2015: 3.9; post-2015: 3.6) and 13.5 among Germans (pre-2015: 13.8; post-2015: 13.0).

The SMR of the POI in the full study period 2000 to 2020 was 0.38 with 95% CI = 0.35–0.41 (Germans: SMR = 1.00; 95% CI = 1.00–1.01). The SMR of the POI for the pre-2015 period was 0.35 with 95% CI = 0.30–0.41, and the SMR of the POI for the post-2015 period was 0.40 with 95% CI = 0.35–0.45. The POI annual SMR remained constant over the study period except for 2015 (Fig. 4).

The age-specific suicide rates for the POI in the pre-2015 period were 3.76 (age: 0–29 years), 7.24 (30–39), 7.80 (40–49) and 6.06 (50+) for males and 0.62 (age: 0–29), 2.41 (30–39), 2.80 (40–49) and 2.98 (50+) for females. The age-specific suicide rates for the POI in the post-2015 period were 5.16 (age: 0–29), 5.25 (30–39), 5.35 (40–49) and 6.07 (50+) for males and 1.41 (age: 0–29 years), 1.82 (30–39), 2.06 (40–49) and 1.07 (50+) for females.

#### Syrian, Afghan and Iraqi populations in Germany

During the refugee movement studied, Syrians (51.2%), Afghans (18.0%) and Iraqis (20.3%) represented the largest groups of the POI. When comparing the pre- and post-2015 periods, the average sizes of the Syrian, Afghan and Iraqi populations increased from 37 308 to 676 152 (+1712%), from 60 193 to 238 157 (+296%), and from 78 740 to 227 222 (+189%), respectively (Fig. 1(a)). Thus, in the post-2015 period, the large majority of the populations studied consisted of individuals with refugee experience.

In total, 118 suicides were recorded among Syrians (pre-2015: 18; post-2015: 100), 127 among Afghans (pre-2015: 44; post-2015: 83) and 83 among Iraqis (pre-2015: 40; post-2015: 43). In the study period, 4 219 500 life years of Syrians (pre-2015: 512 726; post-2015: 3 706 774), 2 231 899 life years of Afghans (pre-2015: 901 185; post-2015: 1 330 714), and 2 440 278 life years of Iraqis (pre-2015: 1 162 333; post-2015: 1 277 945) were calculated. This corresponds to an unstandardised suicide rate (suicides per 100 000 life years) of 2.8 among Syrians (pre-2015: 3.5; post-2015: 2.7), 5.7 among Afghans (pre-2015: 2.0; post-2015: 7.5) and 3.4 among Iraqis (pre-2015: 3.4; post-2015: 3.4).

**Fig. 2:**
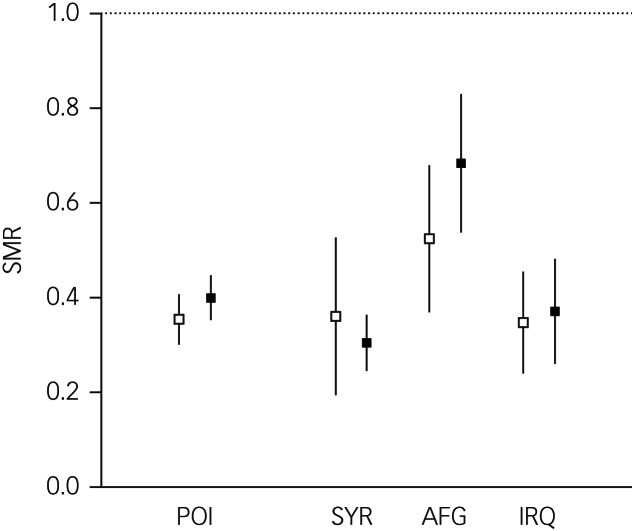
Standardised mortality ratios (SMR) for suicide and 95% confidence intervals for the populations studied. Unfilled squares represent the pre-2015 period and black-filled squares the post-2015 period; POI, aggregated populations of interest; SYR, Syrian; AFG, Afghan; IRQ, Iraqi. Dotted line indicates the SMR of the German reference population.

The SMR for Syrians was 0.36 (95% CI = 0.19–0.53) in the pre-2015 period and 0.30 (95% CI = 0.25–0.36) in the post-2015 period. The SMR for Afghans was 0.52 (95% CI = 0.37–0.68) in the pre-2015 period and 0.68 (95% CI = 0.54–0.83) in the post-2015 period. The SMR of Iraqis was 0.35 (95% CI = 0.24–0.46) in the pre-2015 period and 0.37 (95% CI = 0.26–0.48) in the post-2015 period. The SMR of Syrians, Afghans and Iraqis were lower compared with the German reference population (Fig. 2). In the post-2015 period, between-POI comparisons revealed higher SMR in Afghans compared with Syrians and Iraqis; no differences were found in the pre-2015 period. The pre/post comparison of SMR did not differ for Syrians, Afghans or Iraqis (Fig. 2).

**Fig. 3:**
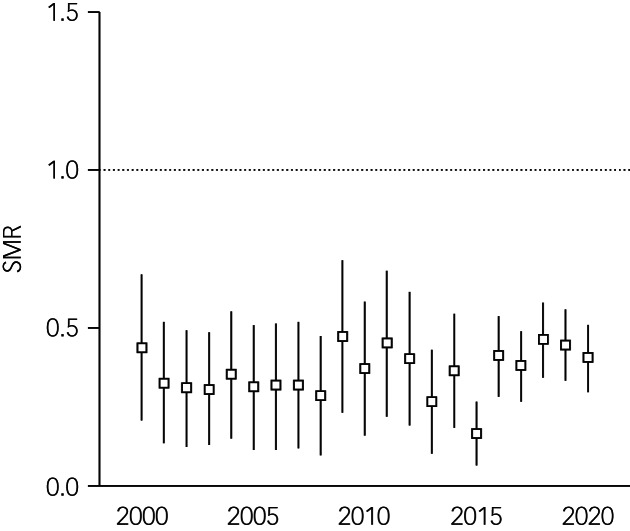
Annual standardised mortality ratios (SMR) for suicide and 95% confidence intervals for aggregated populations of interest. Dotted line indicates the SMR of the German reference population.

**Fig. 4:**
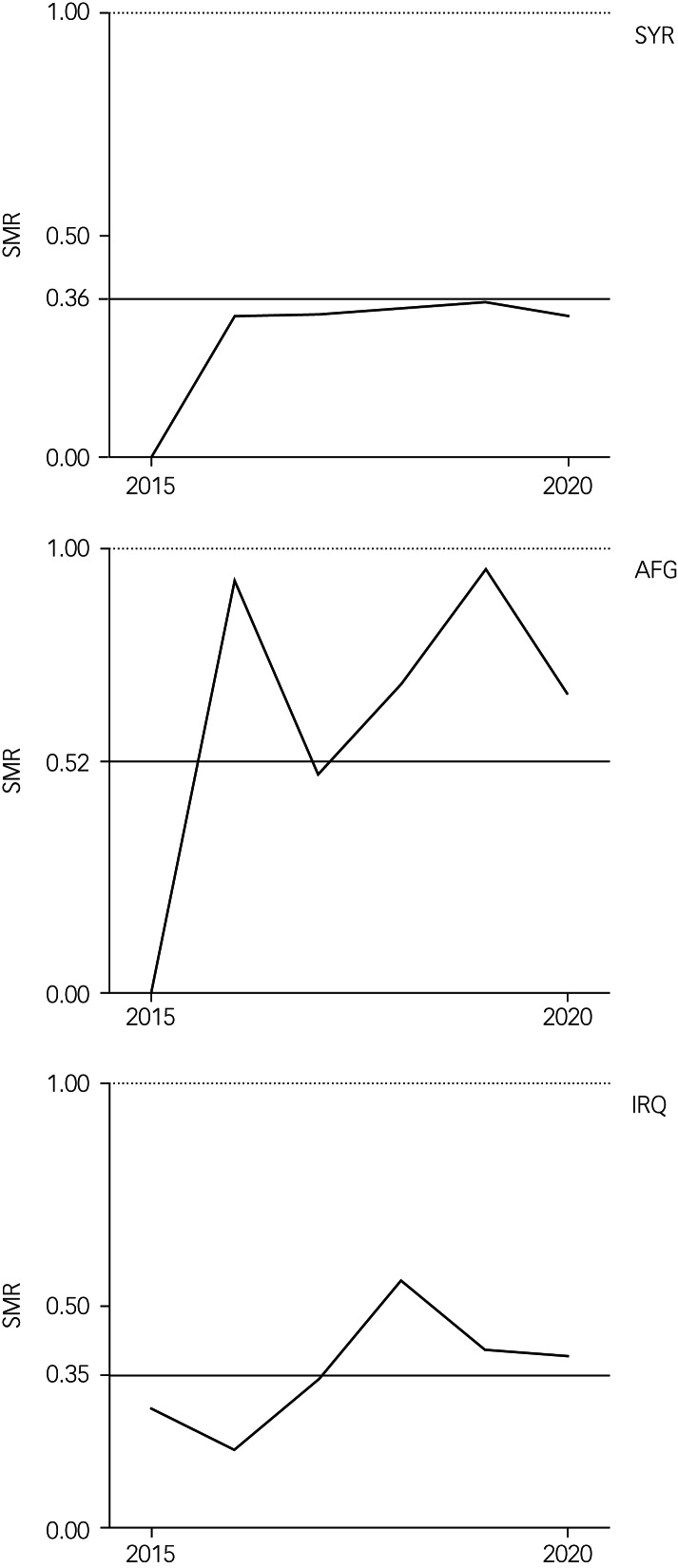
Standardised mortality ratios (SMR) for suicide for the largest populations over time. SYR, Syrian; AFG, Afghan; IRQ, Iraqi. Horizontal line indicates the average SMR of pre-2015 years; dotted line indicates the SMR of the German reference population.

The annual SMR of the aggregated POI are presented in Fig. 3. With the exception of 2015, no changes in suicide risk were found between years. Annual SMR of the Syrian, Afghan and Iraqi populations were calculated for the post-2015 period alone. Annual SMR are plotted for explorative purposes in Fig. 4.

## Discussion

The aim of this study was to investigate the suicide risk among refugees in Germany compared with the German reference population, both before and after the refugee movement of 2015. Our main findings were as follows. (a) The SMR among all immigrant groups in aggregate were consistently lower than that of the German reference population, both before and after the refugee movement in 2015. (b) This pattern also held for the largest populations studied – Syrian, Iraqi and Afghan. (c) No significant differences in refugees’ suicide risk were found when comparing the time periods before and after the refugee movement. (d) The overall SMR among the non-German populations studied remained stable in the years following the refugee movement. (e) After the refugee movement, the SMR among the Afghan population was significantly higher compared with those of the Syrian and Iraqi populations.

Immigration is a multifaceted, complex issue, and there is thus a major risk of overgeneralising research findings. This is also the case for research relating to refugees, as their situation is determined by multiple factors, including reason for flight; age and developmental stage; exposure to physical, sexual or psychological violence; flight within a social network versus social disruption; social status and acceptance in countries of origin and destination; and post-migration factors such as the process of obtaining asylum status or the conditions of (collective) accommodation.^22^ We investigated whether the suicide risk of refugees who arrived in Germany as a result of the flight movement in 2015 differed from that of the population of nationals of the same countries already resident in Germany.

According to our results, immigrants from the countries examined showed lower suicide risks compared with the host reference sample. This was consistent with a recent meta-analysis^16^ that reported a lower suicide risk (SMR = 0.91, 95% CI = 0.90–0.93) in immigrant and refugee populations pooled over 29 studies. A second meta-analysis, focusing on refugees only, reported predominantly lower suicide risk compared with the general population.^23^

A range of register-based studies have investigated the suicide risk of refugees in Sweden.^16,17,19,20,24^ These studies, which were probably based on identical or time-overlapping data-sets, report lower suicide rates among refugees compared with the reference group of individuals born in Sweden. There was no difference in the suicide risk of refugees associated with the country of destination (Norway versus Sweden).^24^ Moreover, the suicide risk of refugees and non-refugee migrants did not differ in a Swedish population.^20^ In the first few years after arriving in Sweden, no suicides were recorded among migrants; in the long term, suicide rates increasingly converged with those of the host country.^20^

Neither of these meta-analyses included post-Arabellion refugees; a high degree of heterogeneity reflects the varying conditions of the studies included. To our knowledge, no previous register-based study has examined the suicide risk of refugees from countries affected by the Arab Spring.

At first glance, our results appears to be consistent with the so-called healthy immigrant effect,^25^ referring to the state of health of immigrants being better than that of the host population, despite a variety of stress factors. This paradox is typically attributed to migration-related selection processes that favour healthier people. The healthy immigrant effect has been studied for various Western destination countries,^26–28^ with a particularly large number of studies on Latin American immigrants to the USA.^29^ However, with regard to mental health, studies of the healthy immigrant effect have reported inconsistent results.^30,31^

It has been well documented that the mental health of post-Arabellion refugees is lower during flight and post migration,^5,6^ which contrasts with the low suicide rates found in this group. Factors such as (a) responsibility for relatives left behind in the home country, (b) gaining back control and (c) experience of self-efficacy, may have a protective effect on suicide rates in post-Arabellion refugees. All of this may contribute to the surprising finding that suicide rates in the post-2015 population characterised by recent refugee experience were similar to the pre-2015 population.

Globally, age-standardised suicide rates in Middle Eastern and North African countries are lower (<5.0) than in Germany (8.3) and than the global average of suicide rates (9.0).^32^ Lower suicide rates have also been reported for Arab minorities in the USA and Germany compared with the general population.^33,34^ Several factors have been suggested that may contribute to the lower suicide rates among Arab ethnic groups. The role of religion as a contributing protective factor is discussed in this context.^35^ Suicide is prohibited across the Arab world, with Islam displaying the most stringent stance against suicide among monotheistic religions. Accordingly, suicide rates in Muslim-majority countries are lower than the global average.^36^ These religious beliefs and attitudes may contribute specifically to a decreased acceptance of suicide in these countries.^35^ It has also been suggested that a collective social focus, strong family ties, affective expressiveness and a positive group ethnic identity may contribute to lower suicide rates among Arab ethnic minority groups.^33,37^

Nevertheless, some countries with a majority Muslim population have higher age-standardised suicide rates than the global average (e.g. Niger, Burkina Faso, Chad).^27^ In the context of suicide risk factors, it is notable that certain factors carry different weights across countries. For example, subjective social status does not seem to play a part in Egypt, and lower religiosity appears less relevant in Morocco compared with, e.g., Algeria and Saudi Arabia.^38^ In Afghanistan, suicide disability-adjusted life years are more highly correlated with substance use in comparison with Middle Eastern and North African countries.^39^ Regarding protective factors, Algeria offers free health services in psychiatric facilities, and the range of services has increased significantly in recent decades,^40^ potentially affecting suicide prevention efforts positively. As important protection and risk factors can remain effective even after an individual's arrival in the destination country, these findings highlight the need for a differentiated and culturally sensitive view of the complex phenomenon of migration. This is supported by suicide rates in the country of origin being associated with immigrant suicide rates in the host country.^34,41^

Among the individual populations examined in our study, individuals from Afghanistan displayed higher suicide risks than those from Syria and Iraq in the post-2015 period (Fig. 2). This pattern was consistent with the ranking of age-standardised suicide rates in the countries of origin (Afghanistan: 6.0 [95% CI 3.4–9.9]; Syria: 2.1 [95% CI 1.3–3.2]; Iraq: 4.7 [95% CI 2.8–7.5]).^32^ Notably, in contrast to the other investigated nations, Afghanistan does not have a stark gender-difference in suicide rates.^32^

Our findings are in keeping with those of another German study reporting that the mental health of refugees from Afghanistan who arrived in Germany between 2014 and 2017 was poorer than that of Syrian refugees.^42^ The same conclusion was reached in a Swedish study.^43^ Potential causes may be country-specific and rooted in the living conditions of the country of origin. For example, about half of the Afghans had already had at least one refugee experience before the 2015 refugee movement, and many of them had been displaced multiple times.^44^ Moreover, Afghanistan has seen almost continuous political unrest and war for about 40 years, and more than 60% of the Afghan population has experienced at least one traumatic event.^45^ These circumstances could confound flight-specific and post-migration factors: Germany began deporting protection seekers from Afghanistan in 2016,^46^ and the protection rate of Afghan asylum seekers and refugees declined from 2016 to 2019,^47,48^ which was not the case for asylum seekers from, for example, Syria. As of 2022, approximately 40% of all unaccompanied minor refugees, who lack the protective factor of family support, arriving in Germany originate from Afghanistan.^49^ In a Swedish study based on 12 suicide cases, this group of unaccompanied minor Afghan refugees showed an eight-fold increased suicide risk compared with the reference population.^50^ This was contrary to our findings and those of other Swedish studies^16,18^ reporting lower hazard ratios for suicide in Afghan, Syrian and Iraqi immigrants and refugees.

Overall, the low suicide rates among immigrants of the studied populations were surprising given the many challenges and life history burdens. Our study approach was not designed to investigate the causes of differential suicide rates among immigrants, but previous studies have reported that risk factors such as language barriers, separation from family, lack of information on the healthcare system, and loss of social status and social network are associated with higher suicidal intent or suicide behaviour in immigrant populations.^51,52^ The following approaches should therefore be taken up by policy makers to improve suicide prevention among immigrants: reducing barriers in the healthcare system, improving mental health literacy and connection to a community of similar cultural background, promoting social integration into the host population, enabling integration into the labour market and other measures that strengthen self-efficacy.^53^ More research efforts are needed to shed light on background factors associated with suicide risk in immigrant populations.

### Strengths and limitations

This is the first study to examine suicides among post-Arabellion refugees in Germany, the largest host country in the European Union. The highly divergent demographics of the nationalities studied were controlled for using indirect standardisation. Although we standardised for gender, we only had overall suicide counts for the POI populations and were thus unable to investigate gender-specific SMR, only suicide rate. This is a shortcoming because of the predominance of male immigrants and refugees and the different known gender ratios of suicide rates in the nations under investigation. Of note, we opted for standardisation against German observed suicides within all calendar years from 2000 to 2020 together. Thus, the German national trend of suicide rates across 2000–2020 was averaged and applied in the same way to the standardisation of both the pre- and post-Arabellion estimates for the POI. The long time series allowed a comparison of the SMR for suicide of the individual nationalities before and after the refugee movement. One limitation of the study was the low level of detail of the register-based data-set. For example, no information was available on the (mental) health, trauma experience, religion and current living conditions of the populations analysed. Owing to data protection regulations in Germany, it was not possible to link the official cause-of-death statistics with healthcare data at an individual level. The establishment of a national suicide register that combines data from the cause-of-death statistics with healthcare and socioeconomic data, would help to overcome this limitation in the future. Moreover, the groups in our study were categorised according to citizenship, which is a simplification of the complex phenomenon of migration. The results of the study only apply to migrants with single citizenship, as migrants with dual citizenship (one of which was German) were assigned to the German reference population.

### Future implications

This study complements previous research focusing on suicidal thoughts or attempts in post-Arabellion refugees. In contrast to these phenomena of the suicide spectrum, the suicide risk of the refugee populations studied was lower than that of the German reference population. Similarly, there was no increase in the suicide risk of these immigrant populations throughout the refugee movement from 2015 onward. This resilience in the face of severe mental distress faced by refugees is both surprising and impressive. Further studies should investigate the protective mechanisms underlying the lower suicide rates.

## Supporting information

Le et al. supplementary material Le et al. supplementary material

## Data Availability

The data used in this study are available from the German Research Data Centre (Forschungsdatenzentrum). All information regarding scientific access can be found at https://www.forschungsdatenzentrum.de/de/zugang.
