# Supplementary material for: Suicide among post-Arabellion refugees in Germany
Source: BJPsych Open. 2024 Oct 25;10(6):e188. doi: 10.1192/bjo.2024.755 (PMC11698212; doi:10.1192/bjo.2024.755)
Supplement: Le et al. supplementary material [file S2056472424007555sup001.pdf]

Supplementary considerations on the use of population statistics

In this study, annual data are used to determine population sizes. A very dynamic population development in combination with only annual census data can lead to a distortion of suicide rates / SMR. We therefore address this issue in more detail.

We assume a dynamically increasing population, as was the case for the POI analysed from 2015 onwards (in the scenario of a largely constant population size, the above-mentioned distortion is not relevant). In the case of ideal population statistics, the number of life years spent would correspond to the grey area under the curve (AUC) (Fig A, top-left: reference).

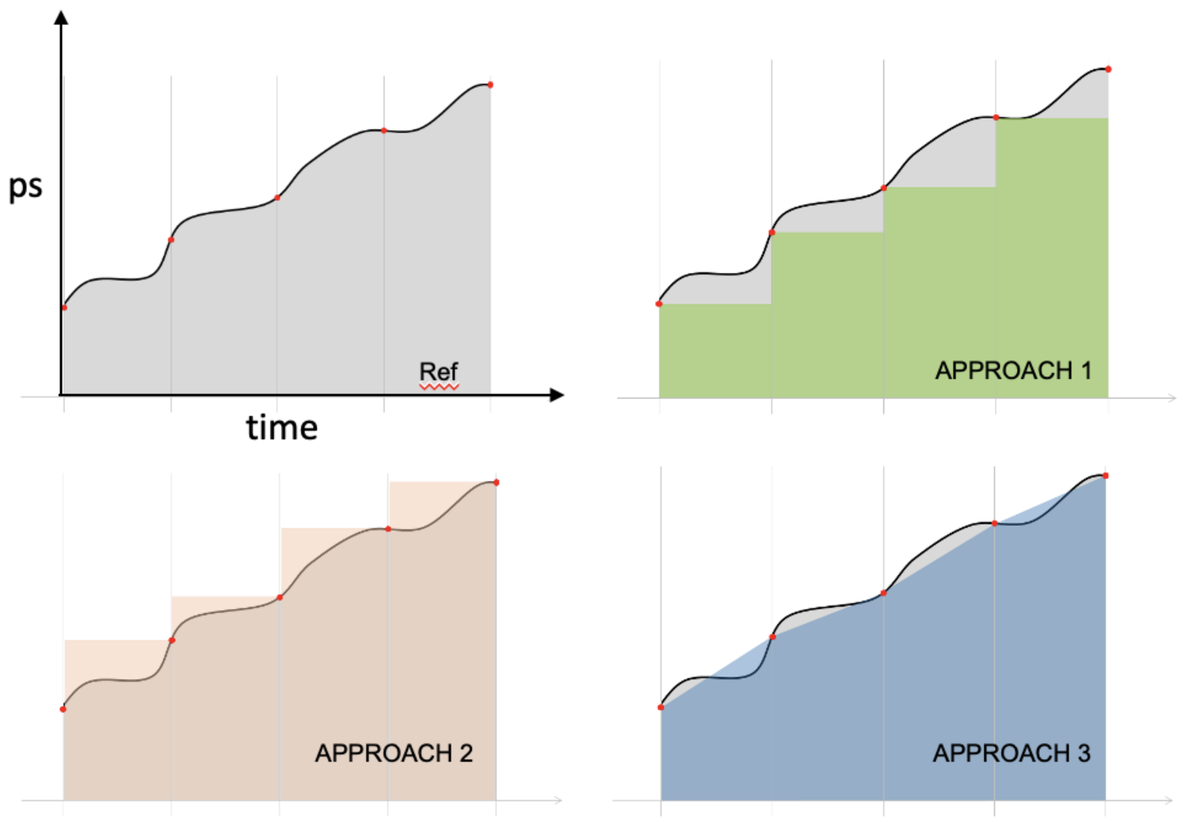

Fig. A
ps = Population size:
Ref = Reference = Ideal population statistics
approach 1 = population census data of the previous year
approach 2 = population census data at the end of the ongoing year
approach 3 = assumtion of the population increasing linear each year = (app1+app2)/2

Approaches to calculate an approximation:

As only annual census data is available for the calculations in our study, all of the following approaches deviate from the ideal population statistics.

Approach 1 is based on the population census of the end of the previous year. It is assumed that a population of this size lived constantly in Germany throughout the index year. This approach underestimates the AUC. The suicide rate (and SMR) is overestimated (as the population size is in the denominator: SR = number of suicides/population size).

Approach 2 is based on the population census of the year just ended. It is assumed that a population of this size has lived in Germany constantly over the index year. This approach overestimates the AUC and underestimates the suicide rate.

Approach 3 assumes a linear population trend between the previous year's census and the census of the year just ended. The population size of approach 3 corresponds to the arithmetic mean of the areas from approaches 1 and 2.

Under the simplifying assumption that the population size was *steadily* increasing during years 2015+, the following conclusions can be drawn:

1. Approach 1 represents the maximum underestimation of the AUC, approach 2 the maximum overestimation of the AUC.

2. The area according to approach 3 is between these extreme points and is therefore closer to the reference, the grey AUC. We have therefore decided to calculate the data using approach 3.

The following explanations aim to apply the three approaches to our data in order to estimate the resulting bias in the SMR.

We calculated the SMR for all three approaches. Figure B shows the effects of the three approaches on the standard mortality ratio of suicide (SMR). The true value lies between approaches 1 (red) and 2 (blue), which represent min and max values. It is

clear that the distortion is less pronounced in the pre-2015 time period (black dots), which is characterised by constant population values. The distortions are more pronounced for the post-2015 time period (white dots), which is characterised by a sharp increase in population sizes.

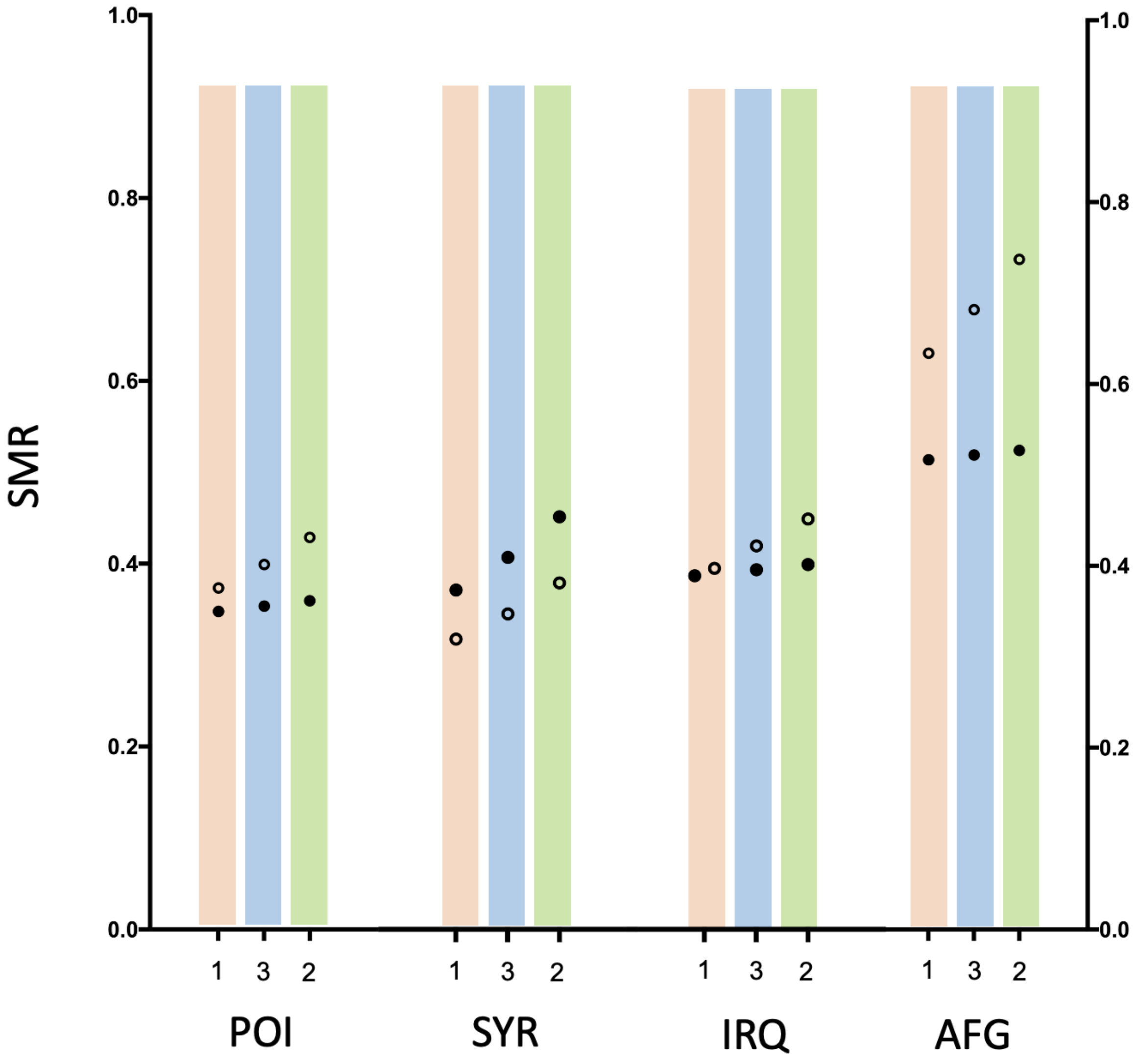

Fig B.
Approach 1 (red), approach 3 (blue), approach 2 (green). POI = Populations of interest; SYR = Syrian; IRQ = Iraqui; AFG = Afghan nationals. Black dot = SMR prä-2015; White dot = SMR post-2015
